# Prevalence of depression among primary caregivers of patients with cancer in Africa: a systematic review and meta-analysis study

**DOI:** 10.3389/fpsyg.2025.1379758

**Published:** 2025-02-13

**Authors:** Yilkal Abebaw Wassie, Belayneh Shetie Workneh, Enyew Getaneh Mekonen, Mohammed Seid Ali, Masresha Asmare Techane, Mulugeta Wassie, Alemneh Tadesse Kassie, Medina Abdela Ahmed, Sintayehu Simie Tsega, Agazhe Aemro, Alebachew Ferede Zegeye, Berhan Tekeba, Tadesse Tarik Tamir, Girum Nakie, Setegn Fentahun, Mamaru Melkam, Getasew Kibralew, Gebresilassie Tadesse, Almaz Tefera Gonete

**Affiliations:** ^1^Department of Medical Nursing, School of Nursing, College of Medicine and Health Sciences, University of Gondar, Gondar, Ethiopia; ^2^Department of Emergency and Critical Care Nursing, School of Nursing, College of Medicine and Health Sciences, University of Gondar, Gondar, Ethiopia; ^3^Department of Surgical Nursing, School of Nursing, College of Medicine and Health Sciences, University of Gondar, Gondar, Ethiopia; ^4^Department of Pediatrics and Child Health Nursing, School of Nursing, College of Medicine and Health Sciences, University of Gondar, Gondar, Ethiopia; ^5^School of Nursing, College of Medicine and Health Sciences, University of Gondar, Gondar, Ethiopia; ^6^Department of Clinical Midwifery, School of Midwifery, College of Medicine and Health Sciences, University of Gondar, Gondar, Ethiopia; ^7^Department of Psychiatry, College of Medicine and Health Sciences, University of Gondar, Gondar, Ethiopia

**Keywords:** primary caregivers, cancer patient, depression, systematic review, Africa

## Abstract

**Background:**

Cancer is one of the main causes of the most extremely stressful events that can elicit emotional reactions such as depression. Cancer patient caregivers are the most involved members of the oncology team and play an important role in patient’s disease management and palliation which may adversely affect their health in the longer run, but many times the caregiver has been overlooked and ignored team. A corresponding systematic review on this topic has not yet been undertaken, even though there have been several studies about depression among primary caregivers of patients with cancer in Africa.

**Methods:**

To find studies, we searched databases such as PubMed, Scopus, Cochrane Library, Science Direct, African Journal Online, and Google Scholar. A Microsoft Excel spreadsheet was used to extract the data, which were then transferred to STATA version 14 for analysis. The statistical heterogeneity was evaluated by using Cochran’s *Q* and *I*^2^ statistics. To identify publication bias, Egger regression tests and funnel plot analysis were used. Sensitivity and subgroup analyses were carried out.

**Results:**

The current systematic review and meta-analysis comprised all 1983 research respondents from 10 studies. The overall pooled prevalence of depression among primary caregivers of patients with cancer was 47.21% with a 95% CI (31.76, 62.65: *I*^2^ = 98.4%). According to subgroup analysis, the pooled prevalence of depression was higher in the studies that used the back depression inventory screening tool (63.95%) (95% CI: 58.76, 69.13). Additionally, we observed a high pooled prevalence of depression in existing studies conducted in Kenya (62.7%) (95% CI: 56.45, 68.95). Finally, a greater prevalence of depression was found among primary caregivers of children with cancer (64.61%) (95% CI: 58.19, 71.03).

**Conclusion and recommendations:**

The current systematic review and meta-analysis showed that depression was prevalent among primary caregivers of patients with cancer in Africa. The study also highlighted variability in prevalence based on country, method of depression assessment, and population subgroups. Therefore, public health interventions targeting the mental health of caregivers should be promoted. Priority should be given to those who care for children.

## Introduction

One-sixth of all fatalities globally are caused by cancer, making it a major public health issue as well as a leading cause of mortality (Jemal et al., 2010). Approximately 10 million deaths in 2020 (Sung et al., 2021) are expected to be related to cancer, of which it is predicted that more than 19.3 million new cases were discovered and identified just already (World Health Organization, 2020). Moreover, 65% of cancer-related deaths worldwide occur in emerging nations (Lin et al., 2021). Sub-Saharan Africa (SSA) countries are frequently listed among the world’s developing nations, and they are currently facing a substantial cancer burden (Ngwa et al., 2022). A little over 70% of cancer-related deaths have occurred in low- and middle-income nations, such as Ethiopia (Chalkidou et al., 2014).

A cancer diagnosis is a terrible and incredibly stressful event for patients and their caretakers. It can cause emotional reactions such as pessimism, worthlessness, guilt feelings, irritability, despair, and depression (Backman, 2013). It has a major impact on the practical, psychological, and physical aspects of the lives of patients and caregivers. As cancer therapy progresses, patients’ needs have become more complex, ranging from tracking their course of treatment and controlling their symptoms to requesting financial/economic, emotional, and psychological support in addition to assistance with physical care (Johansen et al., 2018; Mosher et al., 2013). Family carers often experience changes in their mental, physical, and general wellbeing as well as psychological distress, pressure, and health-risky behaviors as a result of the increased burden. In addition to helping the patient cope with the sickness and treatment, the patient’s family is an important source of support (Vahidi et al., 2016).

More than just consequences to mental and physical health for patients may also be among the possible drawbacks for caregivers. Putting their schooling on hold or quitting altogether is a common occurrence for caregivers, especially younger ones, and can hurt their future earnings and livelihood (Montgomery et al., 2007; Association AP, 2016; Council NR, 2010). 72% of cancer caregivers manage numerous medical or nursing obligations, and they put in an average of 32.9 h per week into their roles. Sixty-two percent of caregivers are categorized as having a “high burden,” and normally, caregivers with cancer have a higher average care load than caregivers without sickness (Maimela, 2016).

The impact of these diseases on those who provide care for cancer patients is significant (Isikhan et al., 2001). Caregiver depression is an emotional illness resulting from the strain of providing care. It can manifest as an inability to perform daily duties, a loss of interest in activities one typically enjoys, and a persistent sadness that lasts for at least 2 weeks. It is a major cause of many diseases and frequently has a detrimental impact on an individual’s quality of life (QOL) (Fekadu et al., 2017). According to several studies, the majority of cancer patients’ caregivers experience caregiver stress and despair (Taleghani et al., 2021).

In underdeveloped nations, almost 50% of individuals suffering from depression do not obtain treatment, contributing to the severity of depression in such regions (Patel, 2007). The frequency of caregiver depression has been documented in the past to range from 20 to 73% (Joling et al., 2015), and higher levels of depression in caregivers are often associated with the care recipient’s behavioral issues, cognitive impairment, functional disabilities, length and volume of care given, caregiver age, with older caregivers being more affected, the caregiver’s sex, with females being more influenced, and the relationship between the caregiver and care recipient, with a sexual relationship having a greater effect (Schulz and Sherwood, 2008; Schulz et al., 1995; Vitaliano et al., 2003; Pinquart and Sörensen, 2003a; Pinquart and Sörensen, 2003b).

Caregivers of cancer patients in Africa face a significant mental health burden, often experiencing depression but lacking access to psychological support within cancer centers. Additionally, the review might reveal specific demographics or situations that put caregivers at a higher risk of depression. This information can be used to prioritize support and develop early intervention strategies for those most vulnerable (Stenberg et al., 2010; Kitrungrote and Cohen, 2006). Currently, there is a lack of data from Africa on caregiver depression. This review can establish a baseline and allow for comparisons with existing global data.

### Research question

What is the pooled prevalence of depression among primary caregivers of cancer patients?

## Methods

### Protocol and registration

The protocol for this systematic review and meta-analysis is registered in the international prospective registration of systematic reviews (PROSPERO) with the ID CRD42024499486. The current review used a Preferred Reporting Items for Systematic Reviews and Meta-Analyses (PRISMA) compliant methodology for searching the literature, selecting studies, extracting data, and reporting conclusions (Page et al., 2021) (Supplementary material).

### Search strategy

A systemic review and meta-analysis was carried out by using research studies that revealed the prevalence of depression among caregivers of patients living with cancer. The databases Scopus, African-Wider, PsycINFO, EMBASE, Google Scholar, Psychiatry Online, World Health Organization (WHO) reports, and PubMed/MEDLINE were used to search for research articles. A list of research that satisfied the eligibility criteria yielded the references. Every database has a unique search method that mixes free texts with controlled vocabulary (also known as caretaker words). The search for these articles took place between 20 November 2023 and 30 December 2023. We used the following search terms: “prevalence” OR “magnitude” or burden,” OR “epidemiology” OR “proportion” OR “incident.” In addition, there are other references to “depression,” “depressive symptoms OR depressive disorder OR major depressive disorder OR major depression,” AND “caregivers,” “carers,” OR “informal caregiver “primary caregivers” or “caregivers” OR “friend caregiver “OR “parent caregiver “OR “cancer patients” OR “caretakers” OR “care providers” OR “family caregivers” AND (“Africa”). Through email communication with the relevant authors, we attempt to get the primary or correspondence author to provide any missing information after the data have been retrieved from the papers. Additionally, the included studies’ reference lists were discussed, and studies based on the study area were not specifically specified.

### Eligibility criteria

#### Inclusion criteria

Articles could be included if they met two requirements: they evaluated the results of interest in primary caregivers, and the main outcome of interest was the prevalence of depression and/or associated factors. The study was designed as a cross-sectional, case–control, and cohort study centered in a community or institution.

#### Exclusion criteria

Previous research reviews, studies on primary caregivers who have a known mental health issue, animal studies, editorials, and studies that only describe depression in paid primary caregivers were disqualified. Additionally, studies whose complete data are unavailable even upon the authors’ request were not included.

### Data extraction

All pertinent data were extracted independently from each publication by YAW, BSW, and EGM using a predetermined data extraction format. A screening tool to assess depression, the prevalence of depression, measures of effect [odds ratio (OR)], confounding variables, and possible associated factors results are all included in the data extraction format. The first author’s name, publication year, the region the study was conducted in, the number of participants, types of tools used to measure depression, and other information were also included. A table divided into two rows was the format used for data extraction. After the searches, SF and GN were cross-checked with each other. When two authors had divergent opinions during the data extraction process, they talked it over until they agreed, at which point they double-extracted the data from other authors. When comparing the observed and expected agreements across writers, either randomly or solely by chance, we have used kappa statistics to illustrate the differences. To determine how reliable the meta-analysis results are, we also performed a sensitivity analysis.

### Outcome measurements

This review aimed to ascertain the pooled prevalence of depression among primary caregivers of cancer patients in Africa. STATA version 14.0 was utilized to calculate the pooled prevalence of depression.

### Quality assessment

For cross-sectional studies, the Newcastle–Ottawa Scale (NOS) (Luchini et al., 2017) was used to evaluate the quality of the research papers included in this review. Compatibility of the included studies, methodological quality of the study, and original article quality in terms of statistical analysis are the components that comprise this quality assessment tool. The original research quality was assessed by each author separately using NOS. The instrument yields a total score of 10, and articles that score 6 or higher on the quality scale were considered medium- and high-quality articles for consideration in this study. If there were disagreements among the authors about the included studies’ quality evaluation, they were resolved by averaging them all.

### Statistical procedure

STATA 14.0 was used for analysis after the data had been extracted and opened in Microsoft Excel. Using texts, tables, and forest plots, the features of the primary studies were displayed. We used the binomial distribution to examine the standard error of prevalence for each primary/original study. We applied a heterogeneity *Q*-test and *I*^2^ test to assess the prevalence of the original research for heterogeneity. The Der Simonian and Laird’s pooled effect of depression was estimated by using a random-effects meta-analysis approach. In addition, we performed a leave-one-out sensitivity analysis to determine the potential cause of heterogeneity in the pooled meta-analysis of Sub-Saharan African primary caregiver’s prevalence of depression. By using Egger’s correlation and Begg’s regression intercept tests at a 5% significant level, publication bias was examined (*x*, *y*). If our analysis reveals publication bias, we formalize the usage of funnel plots, estimate the number and outcome of missing studies, and account for hypothetically absent studies using the non-parametric “trim and fill” approach developed by Duval and Tweedie. To find out how a given group’s characteristics affect the prediction of the pooled prevalence of depression, a subgroup analysis was carried out. To see how removing a single study result from the analysis affects the anticipated pooled prevalence of depression and its conclusion, we conducted a leave-one-out sensitivity analysis. If we found evidence of heterogeneity during analysis, we used the sensitivity analysis outcome to pinpoint its potential cause.

## Results

### Search results

**Figure 1:**
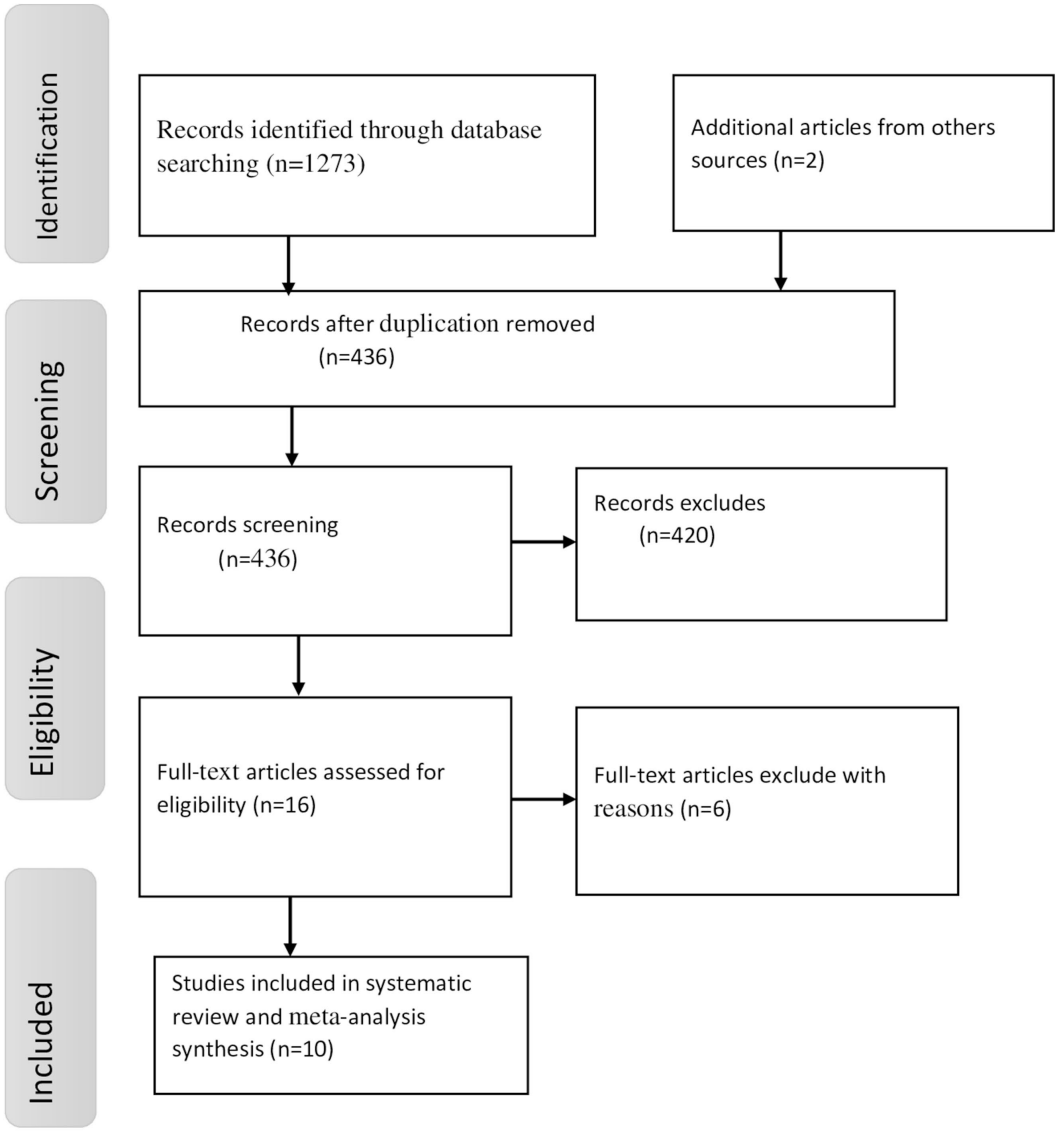
Schematic presentation to show the selection of studies for the systematic review and meta-analysis on the prevalence of depression among primary caregivers of cancer patients in Africa.

A total of 1,275 studies were found for this study using a variety of electronic search techniques. A total of 839 of these studies were eliminated due to redundancy. Additionally, we excluded 420 studies after examining their abstracts and titles because their full texts were unavailable, they were not conducted in Africa, their study demographics and locations differed, and they were irrelevant to our review. Six investigations were eliminated for different reasons after an additional 16 full-text papers were evaluated for eligibility based on the inclusion criteria. Ultimately, this systematic review and meta-analysis contained 10 studies that matched the eligibility criteria (Figure 1).

### Characteristics of included studies

Ten primary research on the prevalence of depression and the factors that are linked to it among primary caregivers of patients with cancer in Africa were included in the current systematic review and meta-analysis. Of those studies, four were conducted from April 2016 to May 2021, while the study time was not disclosed in six studies published between 2013 and 2022. All the studies included in this investigation were cross-sectional study design and five African countries were included. Four articles were conducted in Uganda (Dipio et al., 2022; Katende and Nakamura, 2017; Muliira and Kizza, 2019; Nuwamanya et al., 2023), two articles in Namibia (Tjiroze, 2013) and Tanzania (Malangwa and Mangi, 2022), and the remaining four in Kenya (Adol, 2014; Adol et al., 2020) and Ethiopia (Demissie et al., 2020; Wassie et al., 2022). A total of 1983 study respondents participated, with sample sizes ranging from 65 in Namibia to 421 in Ethiopia. Regarding measurement tools, four studies in Uganda and Ethiopia used the Patient Health Questionnaire-9 (PHQ-9), while three studies in Namibia and Uganda used the Hospital Anxiety and Depression Scale (HADS). The remaining three studies were carried out in Kenya and Tanzania, using the Beck Depression Inventory (BDI) and Hopkins Symptom Checklist 25 (HSC-25), respectively. All original studies included in the current review were completed using a cross-sectional study design. As stated in the included studies, primary caregivers at Uganda Cancer Institute (8.2%) and Tanzania Cancer Center (66.7%) had the minimum and maximum magnitude of depression among primary caregivers of patients with cancer in Africa, respectively (Table 1).

**Table 1:** Characteristics of studies included in this systematic review and meta-analysis on depression among primary caregivers of patients with cancer in Africa.

Authors	Publication year	Country	Measurement tools	Sample size	Prevalence of depression in %	Study population
Tjiroze	2013	Namibia	HADS	65	53.8	Adult
Nuwamanya et al.	2023	Uganda	PHQ-9	366	8.2	Adult
Muliira and Kizza	2019	Uganda	HADS	284	48.2	Adult
Katende and Nakimera	2017	Uganda	HADS	119	26	Adult
Adol et al.	2020	Kenya	BDI	116	62.7	Adult
Adol	2014	Kenya	BDI	114	62.7	Children
Malangwa and Mangi	2022	Tanzania	HSC-25	99	66.7	Children
Dipio et al.	2022	Uganda	PHQ-9	168	46	Adult
Wassie et al.	2022	Ethiopia	PHQ-9	421	45.15	Adult
Demissie et al.	2020	Ethiopia	PHQ-9	231	54.1	Adult

### The pooled prevalence of depression among primary caregivers in Africa

**Figure 2:**
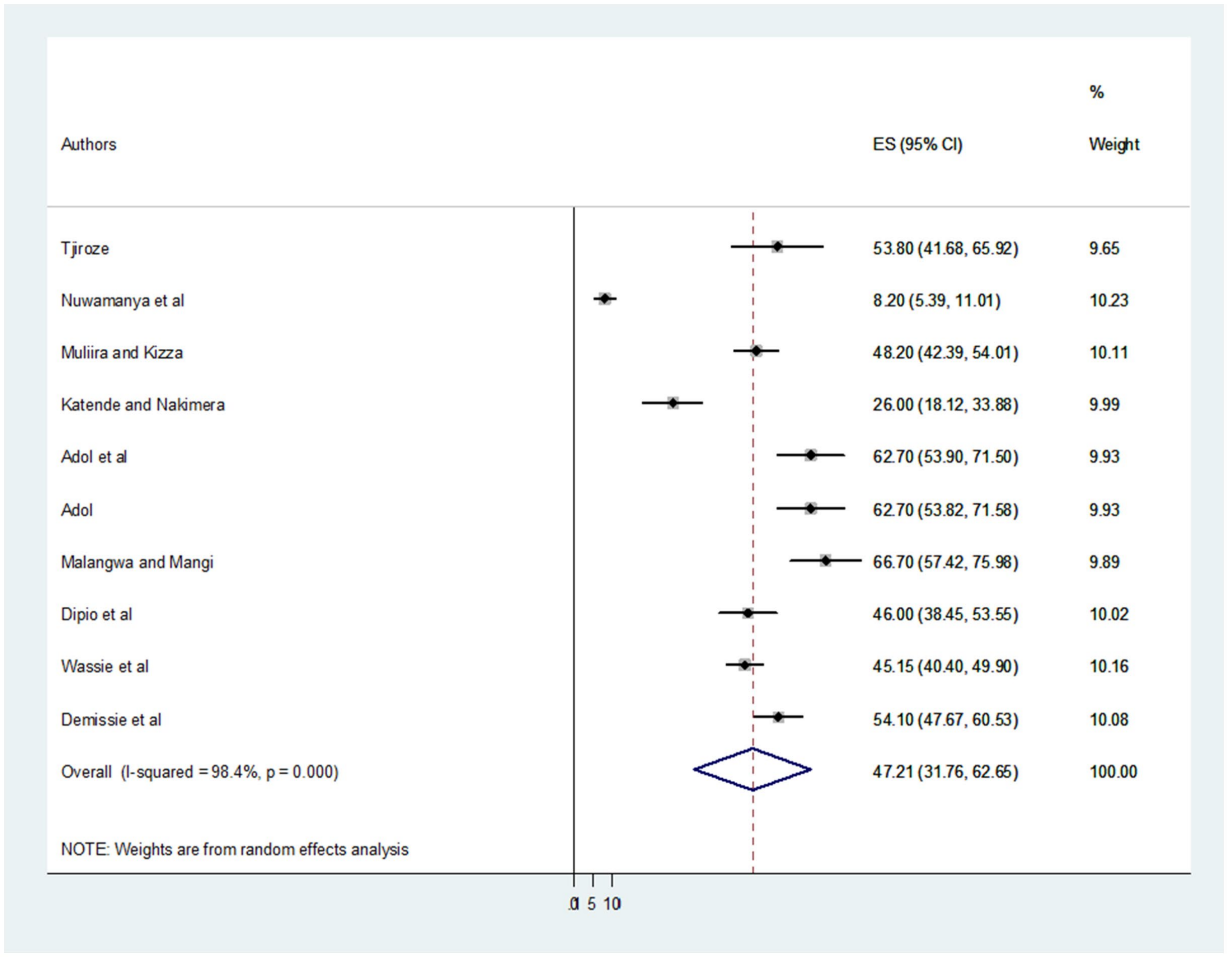
Forest plot showing the pooled prevalence of depression among primary caregivers of patients with cancer in Africa.

To determine the pooled prevalence of depression among primary caregivers of patients with cancer, 10 published publications were included in this systematic review and meta-analysis. The pooled prevalence of depression among primary caregivers of cancer patients in five African countries was found to be 47.21% with a 95% CI (31.76, 62.65) (Figure 2).

### Heterogeneity and publication bias

**Figure 3:**
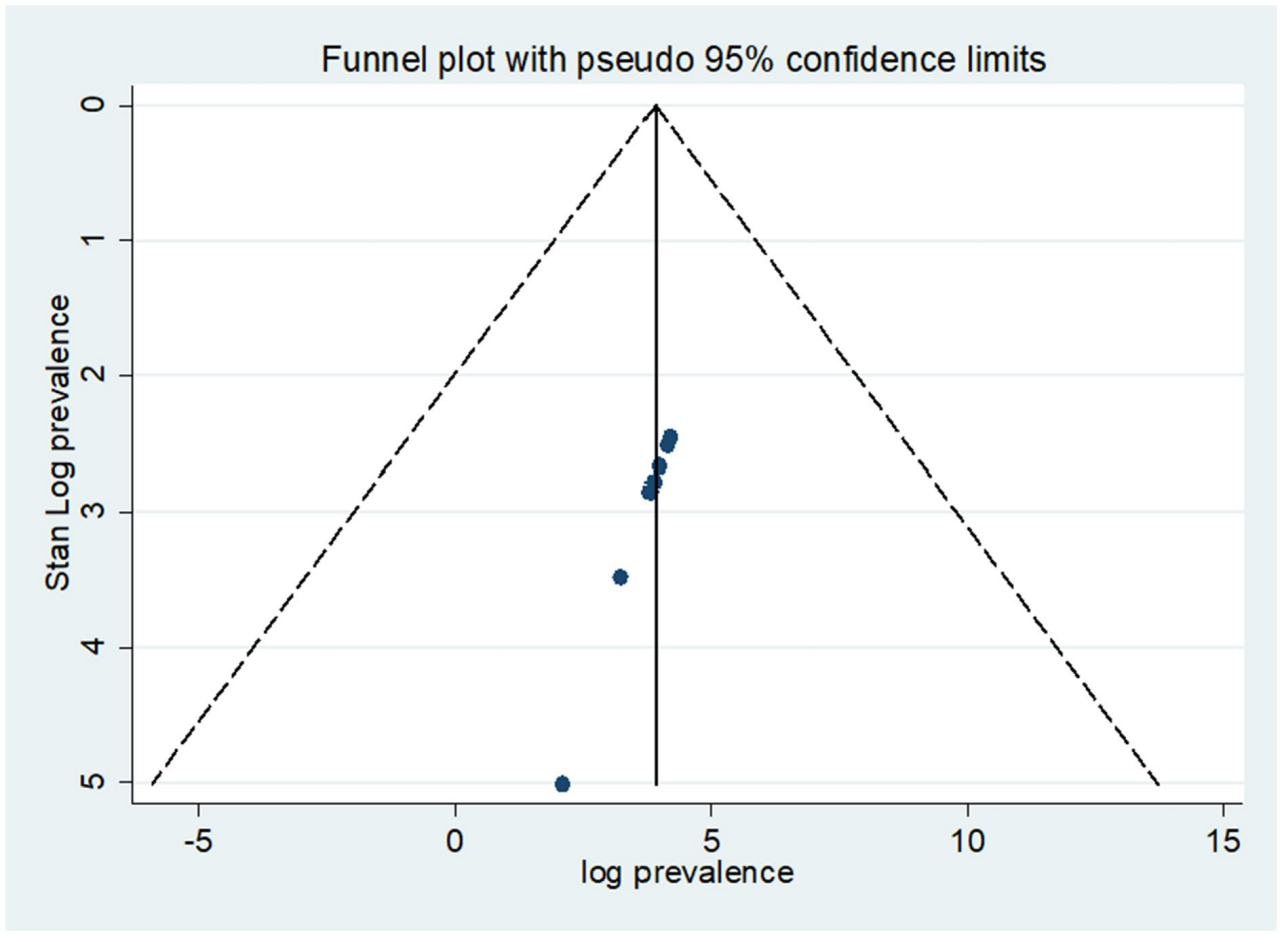
A funnel plot test of pooled prevalence of depression among primary caregivers of patients with cancer.

The included papers in the current systematic review and meta-analysis had heterogeneity, as shown by the test statistics (*I*^2^; *I*^2^ = 98.4%, *p*-value < 0.001). To determine whether there was publication bias in the included papers, two methods were used. The symmetric distribution and lack of publication bias in the included papers were demonstrated by a funnel plot, which was used to verify the first (Figure 3). Additionally, *p* = 0.087 (Table 2) indicates that the Eggers test was used to verify that there was no publication bias.

**Table 2:** Egger’s test of depression among primary caregivers of patients with cancer in Africa.

Std_Eff	Coef.	Std. err.	*t*	*P* > *t*	[95% Conf. Interval]
Slope	−6.321463	9.721601	−0.65	0.534	−28.73951, 16.09659
Bias	14.55263	3.312784	4.39	0.087	6.913341, 22.19193

### Subgroup analysis

A subgroup analysis was performed to identify the possible source of the heterogeneity. The results of the subgroup analysis are shown in Table 3. This review found that there were differences in the prevalence of depression depending on the instruments used to measure depression, the countries where articles were conducted, and the population of study participants. The pooled prevalence of depression among primary caregivers of patients with cancer was different when the methodological differences, especially sensitivity differences of the different screening tools of the studies, were diverse. For example, the subgroup analysis showed that the pooled prevalence of depression in the studies that used the Beck Depression Inventory and Hopkins Symptoms Checklist (63.95) (95% CI: 58.76, 69.13) was higher than those studies that took the patient health questionnaires and hospital anxiety and depression scale screening tools (38.26) (95% CI: 13.03, 63.50) and (42.37) (95% CI: 25.98, 58.76), respectively. Moreover, a comparatively high prevalence of depression across nations was observed; studies were conducted in Kenya (62.7) (95% CI: 56.45, 68.95), followed by studies conducted in Namibia and Tanzania (60.86) (95% CI: 48.28, 73.45). Finally, a higher prevalence of depression was found among primary caregivers of children with cancer (64.61) (95% CI: 58.19, 71.03) than primary caregivers of adult patients with cancer (42.87) (95% CI: 26.28, 39.46).

**Table 3:** Subgroup analysis of depression among primary caregivers of patients with cancer in Africa.

Variables	Subgroup	Number of studies	Prevalence (95% CI)	*I*^2^%	*p*-value
Country	Uganda	4	32.01(9.03, 54.99)	98.5	0.000
	Kenya	2	62.7(56.45, 68.95)	0.00	1.000
	Ethiopia	2	49.35(40.60, 58.11)	79.2	0.028
	Others^*^	2	60.86(48.28, 73.45)	63.5	0.098
Measurement tools	PHQ-9	4	38.26(13.03, 63.50)	99.0	0.000
	HADS	3	42.37(25.98, 58.76)	91.6	0.000
	Others^**^	3	63.95(58.76, 69.13)	0.00	0.782
Study population	Adult	8	42.87(26.28, 39.46)	98.4	0.000
	Children	2	64.61(58.19, 71.03)	0.00	0.542

NB: *:-Namibia, Tanzania; **:-BDI, HSC-25.

### Sensitivity analysis

Sensitivity analysis was used in the current systematic review and meta-analysis to examine the heterogeneity of those studies by carefully removing one author or one study to determine the impact of each study’s findings on the pooled prevalence of depression among primary caregivers of cancer patients. Since all of the figures fell within the expected 95% CI, it can be concluded from the data that the inclusion of one study did not significantly alter the prevalence of this review (Table 4).

**Table 4:** Sensitivity analysis of depression among primary caregivers of patients with cancer in Africa.

Authors	Estimate 95% CI	Heterogeneity	
		*I* ^2^	*p*-value
Tjiroze	46.50 (31.13. 62.87)	98.5	0.000
Nuwamanya et al.	51.43 (44.08, 58.77)	88.8	0.000
Muliira and Kizza	47.11 (30.03, 64.19)	98.5	0.000
Katende and Nakimera	49.57 (32.63, 66.52)	98.5	0.000
Adol et al.	45.49 (29.39, 61.59)	98.4	0.000
Adol	45.49 (29.39, 61.59)	98.4	0.000
Malangwa and Mangi	45.06 (29.12, 60.99)	98.4	0.000
Dipio et al.	47.35 (35.01, 64.19)	98.5	0.000
Wassie et al.	47.46 (29.76, 65.17)	98.5	0.000
Demissie et al.	46.44 (29.85, 63.02)	98.4	0.000

## Discussion

The current study conducted a systematic review and meta-analysis, estimating the African level of pooled prevalence of depression among primary caregivers of patients with cancer showing an interesting result. Ten studies, with 1983 primary caregivers, investigated across five countries, were encompassed in the current review. In general, the review findings indicated that the overall prevalence of depression among primary caregivers of patients with cancer in Africa was found to be 47.21% with a 95% CI (31.76, 62.65). The prevalence of depression found in the current review was consistent with related studies conducted in other countries. According to the previously conducted studies 6 years (Geng et al., 2018) and 2 years (Bedaso et al., 2022) before, the overall global prevalence of depression among primary caregivers of patients with cancer was comparable with our findings of 42.3 and 42.08%, respectively.

It is difficult to identify existing research regarding the general pooled prevalence of depression among primary caregivers of cancer patients because published articles were particular or contained a single finding. The result of this systematic review and meta-analysis found a higher prevalence of depression than the results of other studies conducted in the Midwestern United States 26.9% (Kim et al., 2014), Malaysia 29.4% (Ambigga Devi et al., 2005), and studies conducted in Turkey 29% (Ustaalioğlu and Acar, 2017). The discrepancy between our findings and those of these studies might be because most other studies conducted in different countries had a single finding, whereas this study had a pooled prevalence from different studies. The possible reason may be due to health care delivery policy differences and the country’s priority to cancer treatment and prevention in the case of the Midwestern United States and Turkey (Busse, 2010).

On the contrary, the findings of the current systematic review and meta-analysis were lower than the results of other studies conducted in China (63.5%) (Yang et al., 2012) and Korea (67%) (Rhee et al., 2008). The possible justification for this difference could indicate that as a whole, Korean caregivers are more emotionally perceptive. Rather than expressing their anger when things go wrong, Koreans prefer to repress it (Lee and Farran, 2004). It might be also the Korean government also places a strong emphasis on the value of traditional family values, which mandate that family caregivers handle practically all of the care for their relatives who have chronic illnesses. Family caregivers in Korea bear a great deal of load and distress due to the lack of health and social infrastructures enabling long-term care for chronically ill patients or home health care (Chee and Levkoff, 2001).

According to a subgroup analysis outcome, using the measurement tool employed to recognize the level of depression, has displayed considerable dissimilarity across studies among primary caregivers of patients with cancer. A greater prevalence of depression has been seen in studies conducted by using a back depression inventory screening tool (63.95%), while studies conducted via patient health questionnaire screening tools have shown a lesser pooled prevalence of depression (38.26%). The possible justification for this variation may be due to differences in the psychometric qualities of the measurements used in various research. Moreover, it may be due to that many scales for depression have been developed and validated to screen or measure the severity of depression but every scale has its strengths and limitations as well and every screening tool also varies in the extent of psychometric evaluation (Behera et al., 2017; El-Den et al., 2018).

Furthermore, the findings of the subgroup analysis showed that a high pooled prevalence of depression existed in studies conducted in Kenya (62.7%), whereas a lower prevalence of depression was observed in studies conducted in Uganda. The possible reason for this discrepancy could be due to methodological differences, especially sensitivity differences of the different assessment tools across different nations. Studies conducted in Kenya used a back depression inventory, while studies conducted in Uganda used the hospital anxiety depression scale. It could also be due to different health-related strategies and policies as well, as giving special emphasis on common mental disorders in different nations (Drew and Funk, 2010).

Finally, the results of the subgroup analysis illustrated that a higher prevalence of depression was found among primary caregivers of children with cancer (64.61%) than primary caregivers of adult patients with cancer (42.87%). The probable explanation for this deviation might be because most of the time primary caregivers of children were parents, and the majority of societies perceive and trust parent caregivers to be more caring than other relative caregivers for their children with cancer (Bretones Nieto et al., 2022). Hence, parents try to help the patient wholeheartedly because they feel an extreme commitment toward their child. Moreover, families that have a child with cancer bear heavy financial burdens. The family’s finances were strained by the expense of providing medication, food, hospitalization, and transportation. Additionally, parents and family members bear the burden of indirect expenses such as fewer working hours that translate into lower incomes, a loss of livelihood, and fewer career chances (Pishkuhi et al., 2018).

### Limitations of the study

However, this systematic review and meta-analysis provided important clinical and research advantages. The pooled effect of depression among primary caregivers has the following limitations: first, a limited number of studies were included in this systematic review and meta-analysis, and there was heterogeneity among the included primary articles. Finally, the current study focused on the pooled prevalence, which is challenging to discuss with previous single-finding published studies.

## Conclusion

The current systematic review and meta-analysis showed that depression was prevalent among primary caregivers of patients with cancer in Africa. The study also highlighted variability in prevalence based on country, method of depression assessment, and population subgroups. Therefore, public health interventions targeting the mental health of caregivers should be promoted. Priority should be given to those who care for children.

## Data Availability

The original contributions presented in the study are included in the article/Supplementary material, further inquiries can be directed to the corresponding author.
