# Corrigendum: Prevalence of depression among primary caregivers of patients with cancer in Africa: a systematic review and meta-analysis study

**DOI:** 10.3389/fpsyg.2025.1587534

**Published:** 2025-04-15

**Authors:** Yilkal Abebaw Wassie, Belayneh Shetie Workneh, Enyew Getaneh Mekonen, Mohammed Seid Ali, Masresha Asmare Techane, Mulugeta Wassie, Alemneh Tadesse Kassie, Medina Abdela Ahmed, Sintayehu Simie Tsega, Agazhe Aemro, Alebachew Ferede Zegeye, Berhan Tekeba, Tadesse Tarik Tamir, Girum Nakie, Setegn Fentahun, Mamaru Melkam, Getasew Kibralew, Gebresilassie Tadesse, Almaz Tefera Gonete

**Affiliations:** ^1^Department of Medical Nursing, School of Nursing, College of Medicine and Health Sciences, University of Gondar, Gondar, Ethiopia; ^2^Department of Emergency and Critical Care Nursing, School of Nursing, College of Medicine and Health Sciences, University of Gondar, Gondar, Ethiopia; ^3^Department of Surgical Nursing, School of Nursing, College of Medicine and Health Sciences, University of Gondar, Gondar, Ethiopia; ^4^Department of Pediatrics and Child Health Nursing, School of Nursing, College of Medicine and Health Sciences, University of Gondar, Gondar, Ethiopia; ^5^School of Nursing, College of Medicine and Health Sciences, University of Gondar, Gondar, Ethiopia; ^6^Department of Clinical Midwifery, School of Midwifery, College of Medicine and Health Sciences, University of Gondar, Gondar, Ethiopia; ^7^Department of Psychiatry, College of Medicine and Health Sciences, University of Gondar, Gondar, Ethiopia

**Keywords:** primary caregivers, cancer patient, depression, systematic review, Africa

In the published article, an author name was incorrectly written as Setegn Fentahu. The correct spelling is Setegn Fentahun.

The authors apologize for this error and state that this does not change the scientific conclusions of the article in any way. The original article has been updated.

