# Supplementary material for: Prevalence of depression among primary caregivers of patients with cancer in Africa: a systematic review and meta-analysis study
Source: Front Psychol. 2025 Feb 13;16:1379758. doi: 10.3389/fpsyg.2025.1379758 (PMC11867047; doi:10.3389/fpsyg.2025.1379758)
Supplement: Supplementary file 2 [file Table_2.docx]

Table_S2 Quality assessment of depression and its associated factors in Africa included studies in this meta-analysis and systematic review.

| Author, year of  Publication | Q1 | Q2 | Q3 | Q4 | Q5 | Q6 | Q7 | Q8 | Q9 | Total score (9%) |
| --- | --- | --- | --- | --- | --- | --- | --- | --- | --- | --- |
| Tjirozeet,2013 | Y | Y | Y | Y | Y | Y | Y | Y | Y | 9 |
| Nuwamanya et.al,2023 | Y | Y | Y | Y | Y | Y | Y | Y | Y | 9 |
| Muliira and Kizza,2019 | Y | Y | Y | Y | Y | Y | Y | Y | Y | 9 |
| Katende and Nakimera,2017 | Y | Y | Y | Y | Y | Y | Y | NA | Y | 8 |
| Adol et al,2020 | Y | NA | Y | Y | Y | Y | Y | Y | Y | 8 |
| Adol,2014 | Y | NA | Y | Y | Y | Y | Y | Y | Y | 8 |
| Malangwa and Mangi,2022 | Y | Y | Y | Y | Y | Y | Y | Y | Y | 9 |
| Dipio et al,2022 | Y | Y | Y | Y | Y | Y | NA | Y | Y | 8 |
| Wassie et al,2022 | Y | Y | Y | Y | Y | Y | Y | Y | Y | 9 |
| Demissie et al,2020 | Y | Y | Y | Y | Y | Y | NR | Y | Y | 8 |

**Key:** **Y**= Yes; **NR**= Not reported, **NA**=Not appropriate

**Question codes:**

1. Was the sample frame appropriate to address the target population?

2. Were study participants sampled in an appropriate way?

3. Was the sample size adequate?

4. Were the study subjects and the setting described in detail?

5. Was the data analysis conducted with sufficient coverage of the identified sample?

6. Were valid methods used for the identification of the condition?

7. Was the condition measured in a standard, reliable way for all participants?

8. Was there appropriate statistical analysis?

9. was the response rate adequate, and if not, was the low response rate managed appropriately?
